# Inhibition of Free Radical Polymerization: A Review

**DOI:** 10.3390/polym15030488

**Published:** 2023-01-17

**Authors:** Ibrahim M. Maafa

**Affiliations:** Chemical Engineering Department, College of Engineering, Jazan University, Jazan 45142, Saudi Arabia; imoaafa@jazanu.edu.sa

**Keywords:** runaway polymerization, hazards, inhibitors, synergistic effect, styrene, methyl methacrylate, acrylic acid

## Abstract

Polymerization reactions have caused several severe accidents in the past since they are prone to runaways due to their highly exothermic and auto-accelerating nature. The heat generated during these uncontrolled runaway reactions surpasses the heat removal capacity of the cooling system leading to the auto-acceleration of the reactions. If proper measures are not taken to attenuate this auto-accelerative nature, dangerous consequences ensue, such as rampant boiling of the reaction system fluids or vapor production from secondary reactions. Both these consequences may eventually lead to over-pressurization followed by a thermal explosion. Thus, to eliminate the associated risk, polymerization reactions in industries are carried out in the presence of inhibitors which are injected into the reaction system before the initiation of polymerization. In this review, I have summarized various accidents that have happened in the past due to runaway polymerization implicating that there is an urgent necessity to do further research in this relatively less explored field of polymerization inhibition. To this end, I have completed an exhaustive survey of the various types of inhibitors used in industries and their inhibition mechanisms focusing mainly on the auto-initiated polymerization of styrene, methyl methacrylate, and acrylic acid monomer. Lastly, the synergism in the inhibition performance of a mixture of two types of inhibitors was also compared and discussed in detail.

## 1. Introduction

The reactions in which heat is absorbed (endothermic reactions) are generally more easily controllable than the reactions in which heat is generated (exothermic reactions). Polymerization reactions occurring in the process industries are exothermic in nature. Under the normal operating conditions, the heat released in the reactor is controlled by employing internal jackets and/or cooling coils. When the heat generation rate exceeds the heat removal rate and the control of the reaction temperature is lost, a self-sustaining, uncontrolled runaway reaction may occur. This is because, as the exothermic reaction leads to an increase in the temperature, the reaction rate also increases. The polymerization reactions also lead to the increase in the viscosity due to which the thermal energy exchange and inhibitor spread decrease. Generally, mitigation and control techniques help to remove the generated heat during the reaction while an inhibition technique sways the chemistry of the reaction itself that leads to retardation of the heat-producing reaction mechanism.

Various techniques have been introduced to control the runaway reactions: venting, containment, venting with containment, and reaction inhibition. Out of these techniques, reaction inhibition is a poorly investigated field since runaway laboratory experiments are regarded as highly dangerous [1]. The reaction inhibition technique involves injecting small quantities of a particular chemical substance (inhibitors) into the reactor at the beginning of runaway. The chemical substance employed can entirely suppress the chemical reaction (acting as an inhibitor) or retard the reaction rate to manage time for an emergency response [2]. Additionally, due to the spontaneous formation of undesired polymer or fouling from organic peroxides by auto-advancement of polymerization, the rate of the heat transfer drastically decreases and the time required for cleaning purposes increases [3,4], which makes the handling process less efficient. Thus, the control of undesired polymerization of monomers is a challenging issue faced by industries, and hence inhibitors are necessary to stabilize the monomers during distillation, storage, and transportation processes. 

**Figure 1:**
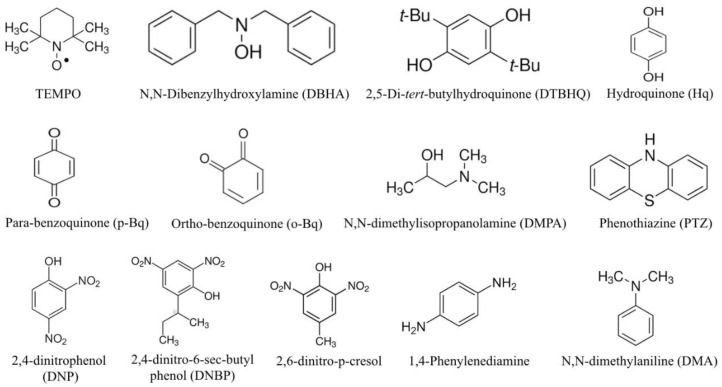
Common inhibitors employed to inhibit the polymerization of styrene, MMA and AA [5].

The inhibitors can act in various ways: (1) they can stop the reaction by deactivating the catalyst, for instance, a small amount of sulfides can deactivate platinum on carbon catalysts; (2) they can terminate a chain reaction via deactivation of the active ends of the spreading chains, thus impeding further proliferation; (3) they can terminate the reaction by imparting a surrogate reaction path with any one of the reactants which is not as active as the usual reaction. The following three kinds of compounds can work as inhibitors: (1) free radicals that are stabilized and can react with the type of radicals that are present in the polymerization system, however, they must not react with monomers. A typical chemical substance in this class is diphenyl picrylhydrazyl (C_6_H_5_)_2_NNC_6_H_2_(NO_2_)_3_; (2) compounds that can bind chemically with primary or polymer radicals producing a new type of radicals that are comparatively non-reactive; (3) compounds that can necessarily behave as transfer agents, although the reaction products of radical displacements are so non-reactive that they cannot re-initiate the reaction. The important types of chemicals that exhibit inhibitory properties are (1) nitro-compounds, (2) compounds that contain sulfur, primarily thiazine, (3) compounds that contain nitrogen, such as nitroso, azo, diazo, etc., (4) aromatic compounds that are based on quinone and require oxygen for activation, and (5) few metal salts and complexes, however, some of them also behave as initiators. In this review article, we surveyed the various type of inhibitors used to inhibit the polymerization of monomer styrene, methyl methacrylate (MMA), and acrylic acid (AA), which are summarized in Figure 1.

To analyze the efficacy of inhibition technique in controlling the polymerization runaway reactions, various pilot plant tests, and laboratory scale trials were performed to polymerize styrene in the presence of benzoyl peroxide [6]. The inhibition technique is befitting to the processes that involve free radicals in the initial phase such as suspension polymerization of methyl methacrylate. The employment of aqueous suspensions allows the chemical reaction to be carried out in water, wherein water acts as a heat dissipation medium. However, due to the presence of water medium various problems arise, such as the particle size homogeneity, suspension stability, and the segregation of the mixture into two phases along with the generation of hot spots in the reactor. Since these phenomena highly depend on the operating parameters, such as reactor temperature and stirring condition, the regulation of suspension polymerization is very important in order to avoid any runaway reaction. Due to the lack of an efficient mitigation measures, uncontrollable boiling of reaction fluids may occur, leading to the over pressurization of the reactor and eventual thermal explosion [7]. A recent investigation of batchwise thermal runaway accidents within the United Kingdom showed that the bulk of such accidents took place in polymerization reactors, accounting for 41 out of 189 accidents from 1962 to 1987 and 10 out of 30 accidents from 1988 to 2013 [8]. Additionally, an incident statistical study by Sales in 2006 reported that 17 out of 132 reactive chemical incidents documented by the major accident-reporting system in the European Commission were induced by polymerization runaway reactions [9].

**Figure 2:**
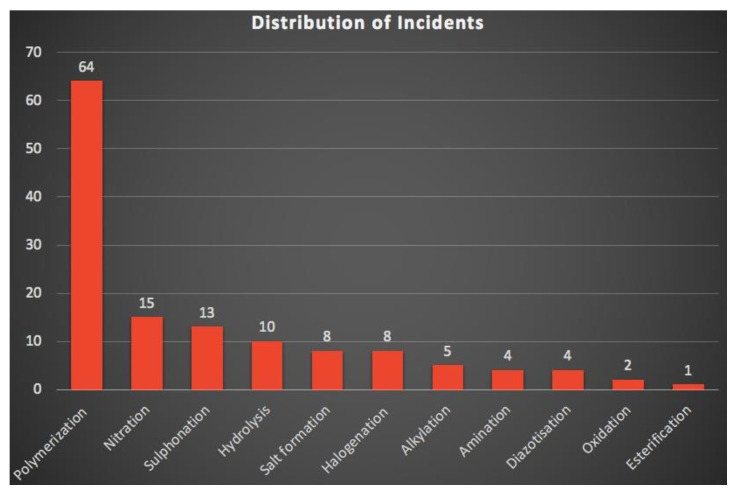
Distribution of incidents that occurred in eleven thermal runaway chemical processes [10].

Among various monomers, styrene is the most extensively used monomer due to its various applications in the chemical industry for the production of polystyrene, acrylonitrile−butadiene−styrene rubber, and other polymers. However, due to the thermal instability associated with monomers, their storage and polymerization processes are susceptible to runaways [10]. Table 1 and Table 2 summarizes the thermal runaway incidents associated with the production and handling of styrene and other chemicals, and only styrene on a global scale, respectively. Figure 2 shows that the highest number of incidents out of eleven chemical processes happened due to the polymerization runaway reactions. 

## 2. Styrene Polymerization Inhibition

Styrene is conventionally an important raw material for the synthesis of polymers, primarily for polystyrene production together with other major commercial co-polymers [17]. It is also extensively used for the production of acrylonitrile–butadiene–styrene (ABS) terpolymer, unsaturated polyesters (UP), emulsion polymers (EP), styrene–acrylonitrile (SAN) copolymer, homopoly (styrene), styrene–butadiene rubber (SBR), elastomer, etc. [18]. Due to its high demand, the production of styrene has significantly increased during the last century [19,20,21]. However, when styrene is distilled, stored, and transported, polymerization chain reaction may auto-advance and extensive heat may be generated [22,23]. The amount of heat generated in the highly exothermic styrene polymerization is around 71 kJ·mol^−1^ [24]. In case the reaction is not properly controlled, then there are risks of deterioration and accidents. 

The control of the spontaneous polymerization reaction of styrene is a challenging task faced by industries due to insufficient knowledge of its chemical reactivity, control systems, and operating methodologies, and the poor design of reactors. Various cases of spasmodic releases, runaway reactions, and subsequent thermal explosion incidents have occurred in the past, which are summarized in Table 3. 

During the last decade, extensive research has been focused on synthesizing inhibitors that are typically added to stabilize and prevent the spontaneous polymerization of styrene. Inhibitors are reactive molecules that terminate the propagation step by reacting with the spreading polymer chains. Adding a very small amount of inhibitors can stop the polymerization chain reaction or significantly decrease its rate. In this review, we present a survey of the recent progress in polymerization inhibition techniques with a focus on styrene monomer polymerization. 

A variety of inhibitors have been investigated in the literature [25,26,27,28], such as phenols that break the polymerization chains by obstructing peroxyl radicals ROO·_2_ [29], aryl amines [30], phenylenediamines [31], N,N-dialkylhydroxylamines [32], m-nitro-p-cresol [33], and 2,6-dinitro-p-cresol [34], and nitroxides such as 2,2,6,6-tetramethyl-4-piperidone nitroxide (I), 4,4′-dimethoxydiphenyl nitroxide (II), and 4,4′-dinitrodiphenyl nitroxide (III) [35]. The most extensively employed inhibitors are (2,2,6,6-Tetramethylpiperidin-1-yl)oxyl or (2,2,6,6-tetramethylpiperidin-1-yl)oxidanyl generally called TEMPO, and their analogues due to their high efficacy and cheap cost [19,20,21]. Among the inhibitors that work even without the presence of oxygen are o-nitrophenols, such as 2,4-dinitrophenol (DNP) and 2,4-dinitro-6-sec-butyl phenol (DNBP).

**Table 3:** Global chemical potential, chemical hardness, electrophilicity, nucleophilicity, and inhibition performance of the neutral Tempo (TempoH) and its various analogues. Reprinted/adapted with permission from Ref. [35]. Copyright 2022, Springer Nature.

Inhibitor	Solvent Used in the Stock Solution	μ^o^	η^o^	ω^o^ (eV)	N^o^ (eV)	Peak Surface Area	% Inhibition
TempoH	Methanol	−1.92	7.62	0.2418	0	467.7	12.5
Amino carboxy	Toluene	−3.04	3.88	1.191	0.75	469.5	12.1
Carboxy	THF (*)	−3.22	3.85	1.351	0.58	358.0	33
Amino	Toluene	−3.05	3.9	1.193	0.73	334.5	37.4
Tempo	Toluene	−3.04	3.89	1.192	0.74	321.2	39.9
Acetamido	Ethanol	−3.22	3.84	1.350	0.59	281	47.4
Butoxy	Toluene	−3.15	3.9	1.272	0.63	270.9	49.3
Oxo	Toluene	−3.45	3.9	1.526	0.33	265.9	50.3
Methacrylate	Toluene	−3.25	3.9	1.350	0.53	249.4	53.3
Methoxy	Toluene	−3.14	3.88	1.270	0.65	241.6	54.8
Benzoate	Toluene	−3.23	3.85	1.359	0.57	206.8	61.3
Hydroxy	Toluene	−3.15	3.9	1.272	0.63	202.0	62.2
Mono radical		−3.14	2.68	1.84	1.25		

### 2.1. TEMPO Inhibitors

**Figure 3:**
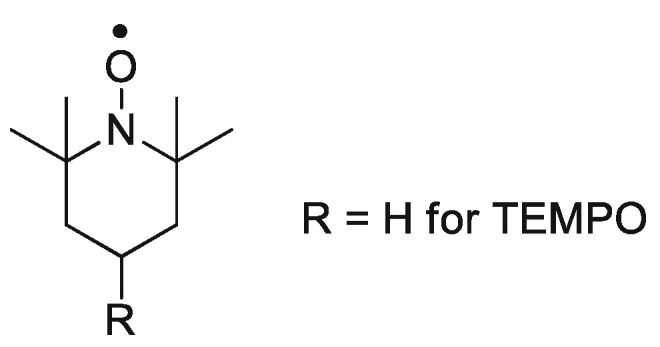
Structure of TEMPO molecule. Reprinted/adapted with permission from Ref. [35]. Copyright 2022, Springer Nature.

The structure of the TEMPO molecule is shown in Figure 3. It is known as (2,2,6,6-Tetramethylpiperidin-1-yl)oxyl or (2,2,6,6-tetramethylpiperidin-1-yl)oxidanyl, and has a formula (CH_2_)_3_(CMe_2_)_2_NO. It is a heterocyclic solid capable of sublimation with a red-orange color.

**Figure 4:**
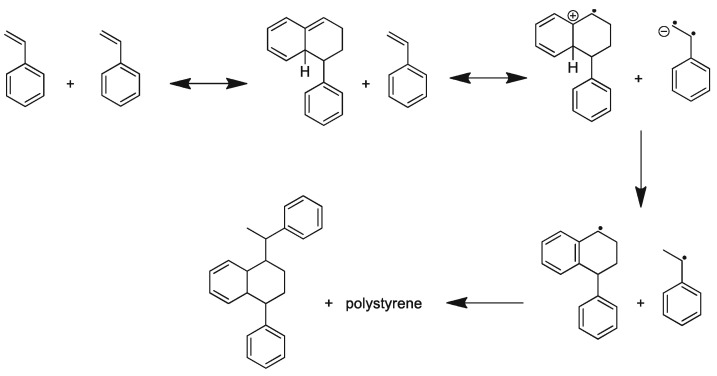
Polymerization without the presence of TEMPO. Reprinted/adapted with permission from Ref. [36]. Copyright 2022, Springer Nature.

**Figure 5:**
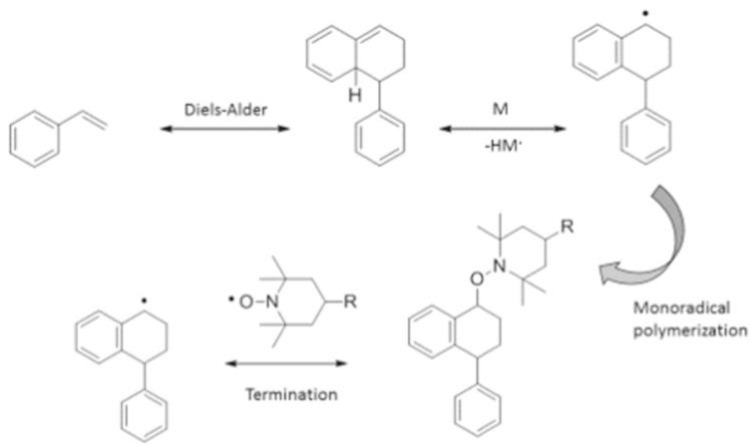
Inhibition mechanism of self-initiated styrene polymerization in the presence of TEMPO inhibitor. Reprinted/adapted with permission from Ref. [36]. Copyright 2022, Springer Nature.

As an inhibitor for polymerization, TEMPO molecules quickly react with carbon-centered radicals to terminate the further propagation of the chain [29], as displayed in Figure 4. Two styrene molecules bind together via Diels–Alder cycloaddition to produce a molecule which can react with another styrene molecule via a one-electron transfer mechanism, producing two benzyl radicals. Without the presence of TEMPO, these benzyl radicals bind with each other or another monomer, thus instigating polymerization (see Figure 5). If TEMPO is present in the reaction system, the benzyl radicals can combine with each other, with TEMPO or with another monomer. However, among all these three reaction possibilities, the reaction of benzyl radicals and TEMPO has the fastest reaction kinetics, and is therefore the most probable [36].

TEMPO is effective in inhibiting polymerization because its molecules do not instigate polymerization or react with another TEMPO molecule, and are sufficiently stable [35]. TEMPO can be employed to either sway the polymerization process in living polymerization reactions or as an inhibitor, depending on the dosage whereby inhibition efficiency increases with the dosage. The utilization of TEMPO in living polymerization reaction systems is an extensively researched topic [36,37,38,39,40,41,42,43,44], however, investigations on the utilization of TEMPO as an inhibitor is inadequately covered by the literature [29]. 

Wadhwa et al. carried out a comparative study of various TEMPO analogues, wherein they gauged the efficacy of substituted groups to inhibit styrene polymerization employing experimental and theoretical methodologies [35]. They explained the experimental results by computing global reactivity indices for the radicals, such as the global electrophilicity index (ω^o^) and global nucleophilicity index (N^o^) via DFT calculations. The global electrophilicity index (ω^o^) can be defined as a base-independent and quantitative metric of Lewis acidity that measures the capability of a molecule to absorb electrons depending on the chemical hardness and chemical potential [45]. The chemical hardness of any chemical species is the resistance offered by the species towards any distortion or polarization of the electron cloud of atom, ion, or molecule [46]. The global nucleophilicity index (N^o^) can be defined as the resistance of a molecule towards electrophilic attack [47] and is described by the following equation:

(1) $$N^{0}=\varepsilon_{\mathrm{HOMO}\left(\mathrm{Nu}\right)}-\varepsilon_{\mathrm{HOMO}\left(\mathrm{TCE}\right)}$$

where $\varepsilon_{\mathrm{HOMO}\left(\mathrm{Nu}\right)}$ is the HOMO energy of the nucleophile within the Kohn–Sham scheme and $\varepsilon_{\mathrm{HOMO}\left(\mathrm{TCE}\right)}$ is the HOMO energy of the TCE (tetracyanoethylene) being considered as reference [48].

**Figure 6:**
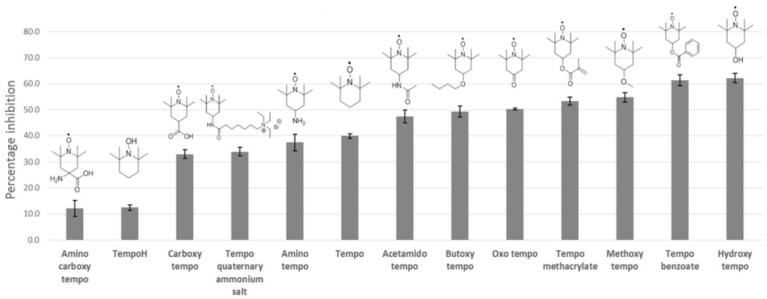
Inhibition efficiency of TEMPO and its analogues. Reprinted/adapted with permission from Ref. [35]. Copyright 2022, Springer Nature.

**Figure 7:**
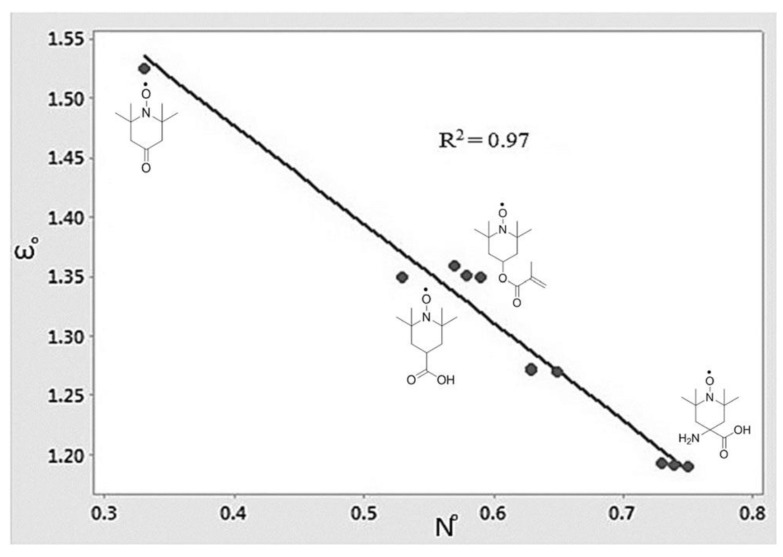
Correlation between ω^o^ and N^o^ of the TEMPO and TEMPO radical analogues reported in Figure 6. Reprinted/adapted with permission from Ref. [35]. Copyright 2022, Springer Nature.

It is evident from Table 4 and Figure 6 that the substituent in the 4′-position in the structure strongly influences the inhibition efficiency of TEMPO, wherein hydroxy TEMPO exhibits the highest inhibition efficiency of 62.2% while aminocarboxy TEMPO exhibits the lowest inhibition efficiency of 12.1%. Additionally, it can be inferred from Table 4 that the substituents of the TEMPO analogues swayed their global electrophilicity (ω^o^) and global nucleophilicity (N^o^). Figure 7 shows that the ω^o^ and N^o^ indices are inversely related to each other which corroborates the previous finding of Vleeschouwer et al. [49]. Table 3 further reveals that the ω^o^ and N^o^ indices of the TEMPO analogues are correlated with the inhibition efficiency, wherein high ω^o^ and low N^o^ correspond to the best inhibitor performers.

### 2.2. Phenolic Inhibitors 

**Figure 8:**
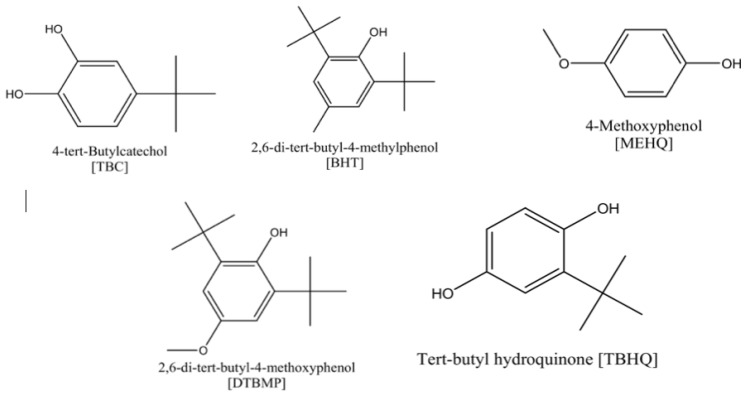
The structure of various phenolic antioxidant inhibitors obtained via DFT [50].

Darvishi et al. studied the influence of phenolic inhibitors and their blends with the previously discussed TEMPO inhibitors on the polymerization of styrene [50]. They employed a combination of experimental and density functional theory (DFT) techniques to compare the activity of these inhibitors. Figure 8 displays the structures of various phenolic inhibitors sketched by DFT.

The various parameters for phenolics obtained from DFT calculations are summarized in Table 4. It should be noted that to inhibit polymerization, the phenolic compounds (antioxidants) should possess low electrophilicity. It is evident from Table 4 that the electrophilicity of phenolic antioxidants follows the following order: MEHQ > TBHQ > TBC > BHT > DTBMP. Therefore, DTBMP exhibits the best inhibition performance to check the undesired polymer’s (UP) formation.

In order to compare the performance of inhibitors, the growth percentage has to be evaluated, which is given by:

(2) $$Growth\;\%=\frac{weight_{final}-weight_{initial}}{weight_{initial}}\times 100$$

where initial weight is the weight of the nucleation source, while the final weight is the weight of UP formed plus the initial weight. It is noteworthy that dimer and oligomer also form in addition to UP. However, the production of these undesired dimers and oligomers is considerably less, due to the application of a radical mechanism which generally results in polymerization. However, the production of undesired dimers and oligomers must be properly controlled within a specified range because they are considered as impurities which do not further react in the polymerization process, and thus, they have to be separated in the purification unit of styrene.

Darvishi et al. performed experiments to investigate the production of dimers and oligomers, and conversion of styrene after 4 and 8 h of operation [50]. Table 5 summarizes the influence of various phenolic inhibitors on the weight of produced, undesired polymer and its growth, the mass fraction of the formed dimer and oligomer, and styrene conversion. It can be seen in Table 5 that the inhibition results remain almost unaltered with the reaction time being increased from 4 to 8 h. Additionally, it is evident from the table that the inhibitor performance of the phenolics follows the following order: DTBMP > BHT > commercial TBC > TBHQ > MEHQ, and DTBMP and BHT are the best performers.

**Figure 9:**
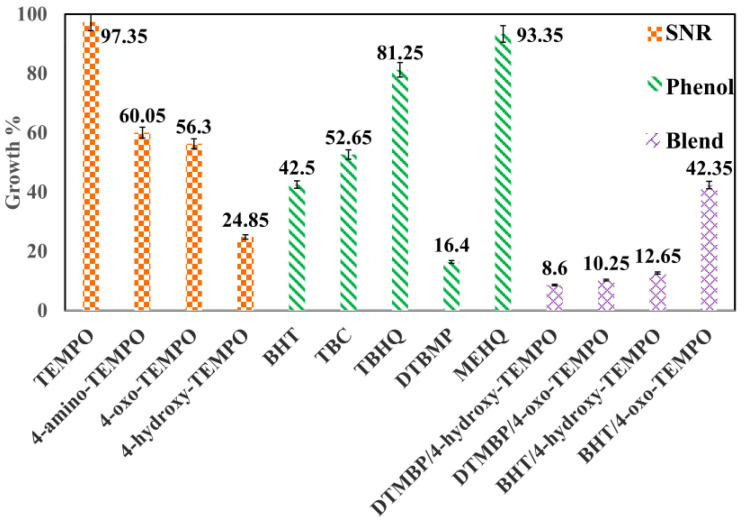
The growth percentage of undesired polymer in the presence of SNRs (TEMPO and its analogues), phenolics, and their blends [50].

Darvishi et al. also studied the synergistic effect of SNRs (TEMPO and its analogues) and phenolics to examine the inhibition performance [50]. They injected a blend of SNRs and phenolics (50 wt.%) in the process with 50 ppm overall concentration. Figure 9 shows the comparison of the growth percentage of various single inhibitors and their blends. It can be inferred from the figure that DTBMP/4-hydroxy-TEMPO exhibits the best inhibition performance, while single component TEMPO exhibits the poorest performance. The synergistic effect of the blends of SNRs and phenolics depends strongly on the hydrogen atom donation and acceptance capabilities of phenolics and SNRs, respectively. By employing the blends of SNRs and phenolics, these capabilities are greatly enhanced, thereby improving the inhibition performance of the blends.

### 2.3. N,N-Dibenzyl hydroxylamine Inhibitor 

**Figure 10:**
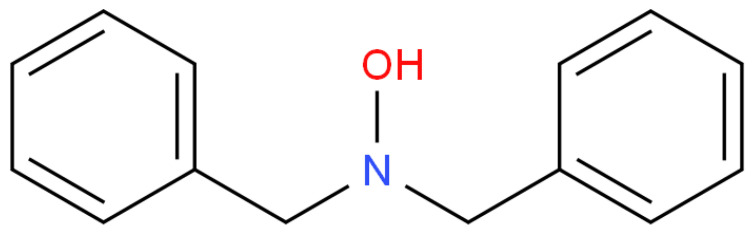
The structure of N,N-Dibenzyl hydroxylamine (DBHA) [53].

N,N-Dibenzyl hydroxylamine, also known as DBHA, acts as an inhibitor for styrene polymerization [51,52]. It is less toxic in nature. The structure of its molecule is shown in Figure 10. Since the RNO–H bond present in the molecule is comparatively weak, this inhibitor can check the propagation of polymerization by hydrogen abstraction. By the homolytic dissociation of the O–H bond of the hydroxylamine, nitroxides (RNO∙) and alkanes (RH) subsequently form, which cannot further propagate the polymerization. 

**Figure 11:**
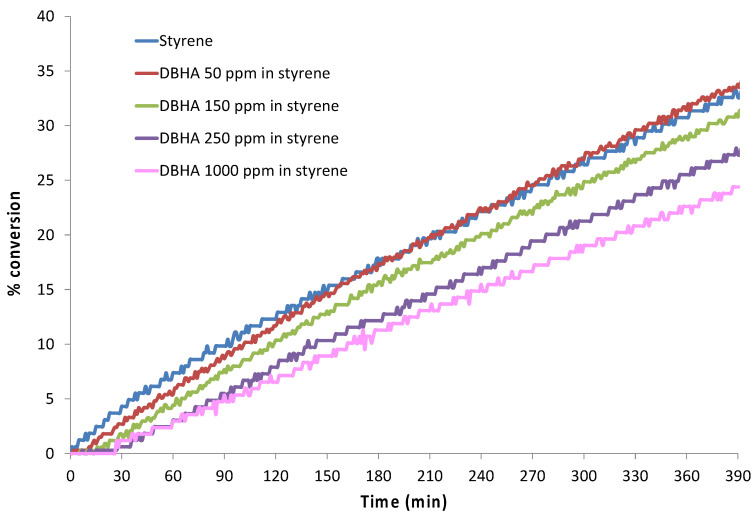
Plots of percentage conversion during the auto-initiated styrene polymerization in the presence of DBHA inhibitor [54].

**Figure 12:**
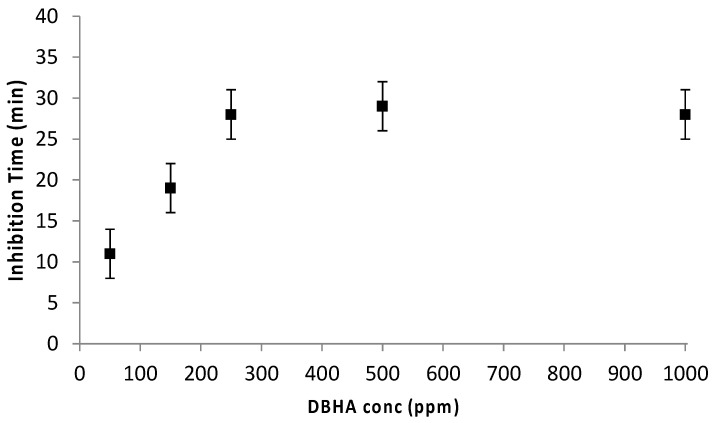
Plot of inhibition time versus DBHA concentration showing a non-linear relationship [54].

Chiara examined the inhibition efficiency of DBHA in the thermal polymerization of air-saturated styrene solution [54]. The author tracked the inhibition process via a dilatometry technique. Dilatometry is a thermo-analytical technique for examining the contraction or expansion of a liquid due to temperature change or occurrence of a chemical reaction. In principle, polymer is denser than monomer due to which a volume change occurs during polymerization process. Thus, if the polymerization is entirely inhibited, no volume change occurs, while during polymerization, a volume reduction takes place. The time during which volume change is not observed is considered as the inhibition time. In this study, the author employed various concentration samples of DBHA in styrene which were sealed in capillary tubes and submerged in an oil bath for 6.5 h at 110 °C. The plots of percentage conversion versus time are shown in Figure 11. The figure displays an initial horizontal line corresponding to the polymerization inhibition period. In this period, the inhibitor captures almost all the produced alkyl radicals that results in the termination of chain polymerization. This inhibition period is typically followed by a retardation period. In this retardation period, the inhibitor is able to capture only a fraction of initiation radicals resulting in partial polymerization. Additionally, during this period the line is not horizontal, however, the slope is smaller than that of the reference line corresponding to pure styrene without the inhibitor. Figure 12 displays the non-linear relationship between the inhibition time and the inhibitor concentration. It is evident from the figure that as the DBHA concentration increases from 50 ppm to 250 ppm, the inhibition time also increases from 11 ± 3 to 28 ± 3 min. However, beyond this concentration, the inhibition time becomes saturated and no further change in the inhibition time was observed. 

### 2.4. 2,5-Di-tert-butyl-hydroquinone Inhibitor

**Figure 13:**
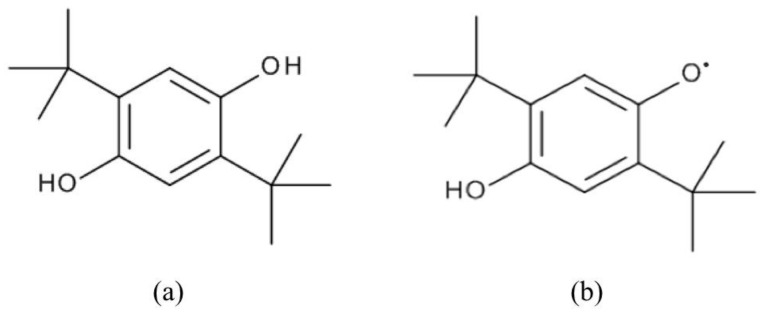
The structure of (**a**) 2,5-Di-tert-butyl-hydroquinone and (**b**) 2,5-Di-tert-butyl-semiquinone [56].

**Figure 14:**
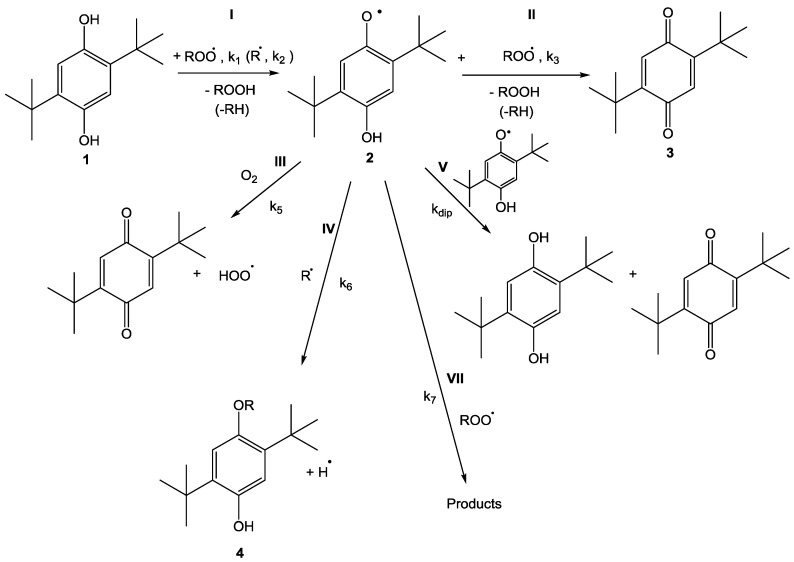
Possible reaction pathways of 2,5-di-tert-butyl-hydroquinone (2,5-DTBHQ) [54].

Figure 13a shows the chemical structure of 2,5-Di-tert-butyl-hydroquinone (2,5-DTBHQ). It is a very effective inhibitor (antioxidant) for styrene polymerization and therefore is extensively utilized in industry. Antioxidants usually function in the presence of oxygen and terminate the reaction chain by supplying hydrogen atoms to alleviate peroxyl radicals (see Reaction I of Figure 14). The bond dissociation energies (BDE) of O-H bond in 2,5-DTBHQ and 2,5-di-tert-butyl semiquinone (see Figure 13b) are 81.2 and 59.1 kcal/mol, respectively [55]. Due to these low BDEs, hydroquinones are very reactive towards peroxyl radicals.

Valgimigli et al. proposed a mechanism wherein semiquinone (2) reacts with molecular oxygen (Reaction III of Figure 14) to produce 2,5-di-tert-butyl-1,4-benzoquinone (2,5-DTBBQ) and hydroperoxyl radical (HOO∙) [57]. The semiquinone (2) terminates a second propagation chain via either hydrogen abstraction (Reaction II) or addition reaction (Reaction IV) and these reaction pathways play a very critical role in the inhibition process. The semiquinone can also quickly disproportionate to 2,5-di-tert-butyl-1,4-benzoquinone (2,5-DTBBQ) (3) and 2,5-DTBHQ through the pathway of Reaction V without any quenching with the peroxyl radical. Through Reaction V, semiquinone regenerates 2,5-DTBHQ which can further react in the system. There is another probability, in which the additional reaction of semiquinone (2) with another peroxyl radical is also possible (Reaction VII).

**Figure 15:**
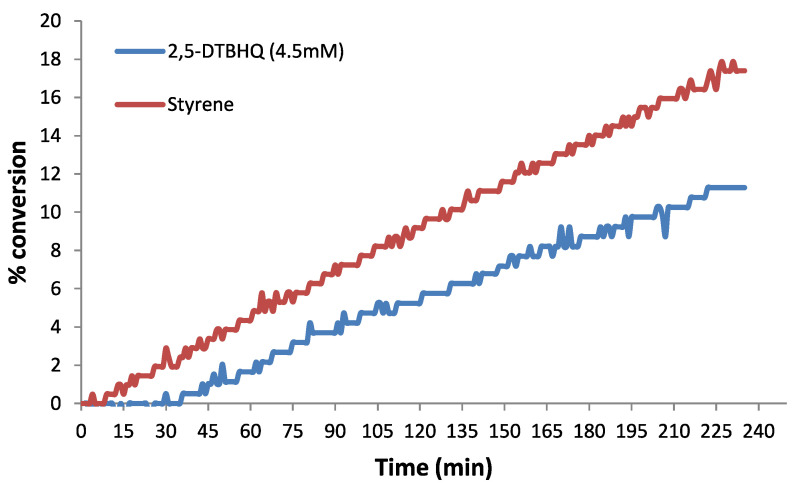
Dilatometry analysis for the inhibition of the thermal styrene polymerization by 2,5-DTBHQ [54].

Figure 15 shows the dilatometry trace of 4.5 mM solution of 2,5-DTBHQ in styrene being analyzed at 110 °C for 4 h to inhibit the thermal styrene polymerization. It is evident from the figure that the inhibition time of styrene polymerization by 2,5-DTBHQ is 36 min, which is in fact worse taking into account the high concentration of the inhibitor. It can be assumed that 2,5-DTBHQ ceases to work in the absence of oxygen and the inhibition time is basically the time taken by the reaction system to become deoxygenated. Thus, the dilatometry trace concludes that 2,5-DTBHQ exhibits an inhibition property only in the oxygenated environment and its efficacy owes to the termination of the propagation chains via hydrogen abstraction. The inhibition phase is then followed by a duration of a little retardation of the styrene polymerization by 2,5-DTBHQ, which can be inferred from Figure 14 in which the slope of the dilatometry trace is smaller as compared to that of polymerization in the absence of the 2,5-DTBHQ inhibitor. More specifically, the formed 2,5-DTBBQ may react with the carbon-centered radicals via the addition reaction and terminate some propagating chains [58]. 

## 3. Methyl Methacrylate Polymerization Inhibition

Ampelli et al. investigated the free-radical polymerization of methyl methacrylate (MMA) in the presence of two types of inhibitors: hydroquinone (Hq) and 1,4-benzoquinone (Bq) [2]. These inhibitors when injected into the reaction system react with the initiator and/or propagation radicals and rapidly decrease the polymerization rate. They performed two set of preliminary experiments to investigate the reaction inhibition of MMA by employing (1) a differential scanning calorimeter (DSC) for bulk polymerization, and (2) a small glass isoperibolic reactor (100 mL) for emulsion polymerization. The emulsion polymerization of MMA was then performed under isothermal batch conditions in a jacketed, stirred stainless-steel reaction calorimeter (2-l). All these experiments were monitored on-line by the divergence criterion that enables the determination of the conditions that can result in a runaway reaction. 

**Figure 16:**
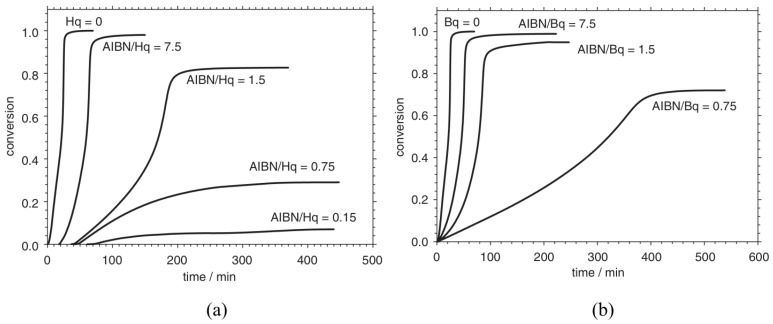
Conversion profiles for DSC experiments in the presence of (**a**) hydroquinone (Hq) and (**b**) 1,4-benzoquinone (Bq). Reprinted/adapted with permission from Ref. [2]. Copyright 2022, Elsevier.

Figure 16a displays the conversion profiles with respect to time for the DSC experiments in the presence of the Hq inhibitor and radical initiator $\alpha ,\;\alpha^{\prime }–$azoisobutyronitrile (AIBN). It can be inferred from the plots that the introduction of the Hq inhibitor results in an inhibition period, the length of which depends on the Hq concentration. Hq acts as an inhibitor since it consumes the free radicals produced by the initiator. In the case of an AIBN/Hq ratio greater than 1 and after the inhibition period, the conversion profile has the same trend as that of the reaction in the absence of the inhibitor. Similarly, the conversion profiles for DSC experiments performed in the presence of the Bq inhibitor is shown in Figure 16b. The experiments do not show any inhibition period and the reaction progresses comparatively slowly reach a low final conversion. Additionally, BQ acts as a retarder since it partially neutralizes the radicals primarily targeting the active centers of the polymer chains.

**Figure 17:**
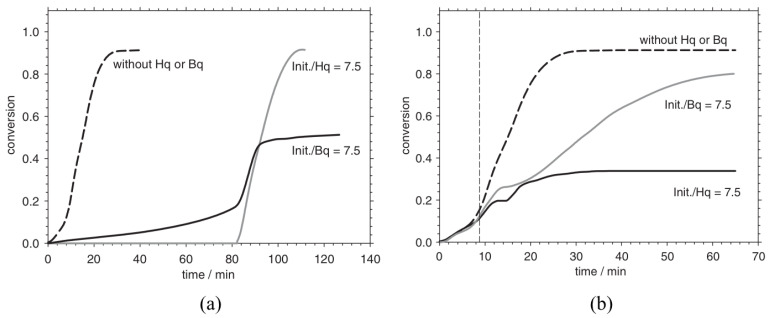
Conversion profiles of the polymerization reaction in 2-1 reaction calorimeter in the presence of hydroquinone (Hq) and 1,4-benzoquinone (Bq) (**a**) at the start of the reaction and (**b**) at the first alarm produced by EWDS. Reprinted/adapted with permission from Ref. [2]. Copyright 2022, Elsevier.

Figure 17 displays the conversion profiles with respect to time for the experiments performed in a stainless-steel reaction calorimeter (2-1). Figure 17a corresponds to the introduction of the inhibitor and radical initiator at the start of the reaction, while Figure 17b corresponds to the introduction at the first alarm given by the early warning detection system (EWDS). The figures suggest that the performance of both the inhibitors is akin to the bulk polymerization processes. The reaction exhibited an initial inhibition period when Hq was injected into the reactor at the start of the reaction and the reaction then progressed at a comparatively higher rate than the standard reaction (without Hq or Bq). When Hq was injected at the first alarm, the runaway reaction rapidly came to an end. However, Bq acted as a retarder and when added at the start of the reaction, the conversion increased slowly and became saturated at a lower value than that of Hq. When Bq was added at the first alarm, the runaway reaction temporarily ceased and then restarted again, reaching a conversion value higher than that of the reaction stopped with Hq, as shown in Figure 17.

Shushunova et al. investigated the kinetic characteristics of the bulk polymerization of MMA under the synergistic effect of inhibitors, such as ortho-benzoquinones and tertiary amines, namely, N,N-dimethylaniline (DMA) and N,N-dimethylisopropanolamine (DMPA) [59]. The kinetics of polymerization were investigated via dilatometry analysis and AIBN was used as an initiator. Two different set of experiments were performed (1) in dark mode at a temperature of 60 °C and (2) under preliminary irradiation. The polymerization inhibition in the irradiation mode was modelled by employing preliminarily irradiated solutions of amines and quinones in MMA. The thermal polymerization of MMA is inhibited when the solutions of quinones and amines in MMA was preliminarily irradiated with visible spectrum.

**Figure 18:**
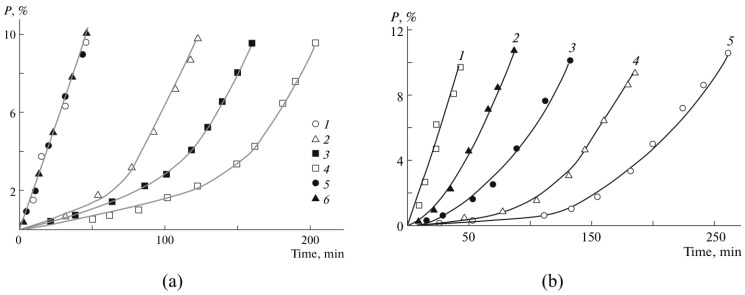
Kinetic plots for MMA polymerization in the presence of: (**a**) BQ-1-DMA mixture: (DMA) = 0.21 mol/L and (BQ-1) × 103 = 0 *(1)*, 4.25 (*2*), 6.38 (*3*), and 8.5 (*4*) mol/L; (DMA) = 0 and (BQ-1) = 4.25 × 103 mol/L (*5*); without inhibitors (*6*), (**b**) BQ-1-DMPA mixture: (DMPA) = 0.21 mol/L and (BQ-1) × 103 = 0 (*1*), 2.12 (*2*), 4.25 (*3*), 8.5 (*4*), and 12.75 (*5*) mol/L. The mode is preliminary irradiation with (AIBN) = 1.14 × 10–2 mol/L and temperature= 60 °C. Reprinted/adapted with permission from Ref. [59]. Copyright 2023, Pleiades Publishing, Ltd.

Table 6 summarizes the experimental data of Shushunova et al. for the variation of the MMA polymerization rate with the concentration of benzoquinones and amines in dark mode. The rate of polymerization of MMA in the absence of the quinone and amine inhibitor was found to be 3.4 × 10^4^ mol/(l s) and no induction period in the polymerization was observed. It is evident from the table that within the considered concentration ranges of BQ-1 (3,6-Di-*tert*-butylbenzoquinone-1,2), BQ-2 (4,5-dimethoxy-3,6-di-*tert*-butylbenzoquinone-1,2), BQ-3 (4-methoxy-3,6-di-*tert*-butylbenzoquinone-1,2), BQ-4 (4-chloro-3,6-di-*tert*-butylbenzoquinone-1,2), and BQ-5 (4,5-difluoro-3,6-di-*tert*-butylbenzoquinone-1,2) mixtures with DMA and DMPA, the MMA polymerization rate to some degree depends on their concentrations. This dependence increases with an increase in the concentration of quinone. However, Shushunova et al. observed retardation in the polymerization reaction when it was carried out under the preliminary irradiation condition [59]. More specifically, they observed well-defined induction periods on the kinetic plots of MMA polymerization being performed in the presence of BQ-1−DMA and BQ-1−DMPA inhibitor mixtures, as shown in Figure 18. It is evident from the curves *1*, *5*, and *6* of Figure 18a and curve *1* of Figure 18b that both the systems exhibited a synergistic effect because when the quinones or amines inhibitors are used alone there is no impact on the kinetics of polymerization.

## 4. Acrylic Acid Polymerization Inhibition

Acrylic acid (AA) is a very reactive vinyl monomer and also the simplest unsaturated monocarbonic acid. It is primarily employed for the production of washing agents (6%), superabsorber polymers (31%), and acrylic esters (53%), while the remaining 10% is employed for special applications [60]. The global demand for acrylic acid was estimated to be 5800 thousand tons in 2021 and is forecast to grow at a compound annual growth rate of 4.18% for the period of 2021–2030 [61]. However, there is a serious problem with its spontaneous polymerization during production, transport, storage, and reprocessing [62]. Due to the highly exothermic polymerization reaction involving reaction enthalpy of −76 kJ mol^−1^, various safety guidelines are required to be strictly followed [62]. The release of reaction enthalpy heat can result in deflagrations and explosions, while the spontaneously produced polymers can cause blockages leading to interruptions, high costs, and loss of production. Therefore, polymerization inhibitor is generally added to the acrylic acid monomer while shipping and storage to avoid its spontaneous polymerization. Among the various inhibitors, PTZ (phenothiazine) and MEHQ (monomethyl ether hydro-quinone) are extensively employed in the chemical industries [63]. During the polymerization of AA monomer at industrial scale, the already present inhibitor may not be removed. Additionally, the dissolved oxygen present in the solution acts as a strong inhibitor [64,65]. Levy reported that the inhibition efficiency of MEHQ was very weak in the absence of oxygen [66]. However, the inhibition efficiency of PTZ is much higher than MEHQ and is independent of the present oxygen level [66,67].

Poly(acrylic acid) is produced at a commercial scale in a semi-batch reactor, wherein the monomer, initiator, and/or neutralizer is continuously fed into the reactor. Li et al. performed simulations to study the semi-batch process employing the same protocols as that for the batch reactor [63]. The process was initiated with an empty reactor and the feed flows of monomer, initiator, and neutralizer were continuously monitored to maintain the ratio of flow rate constant during the entire reaction duration. The feed time in the reactor was 40 min. The reaction was further carried out for 100 min post-feed interval. Thus, the total reaction was 140 min which is consistent with the batch process.

**Figure 19:**
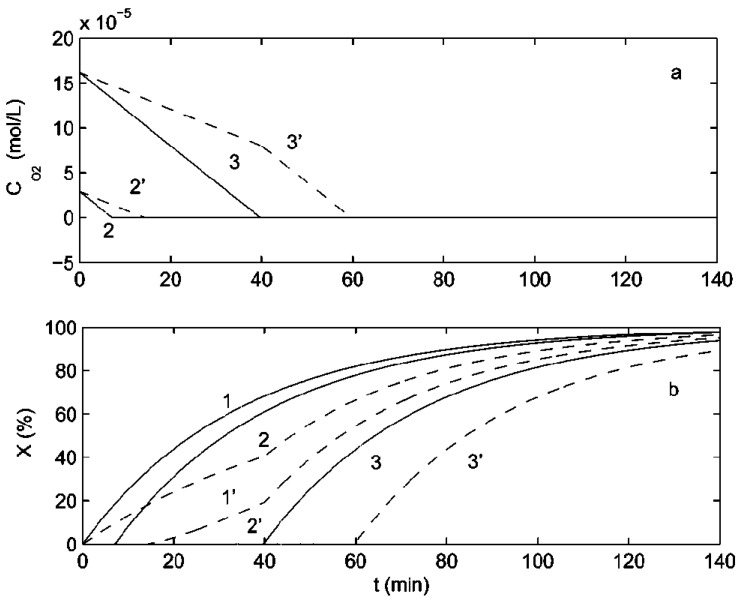
Comparison of (**a**) oxygen and (**b**) monomer conversion profiles in a batch and a semi-batch reactor at three oxygen levels. Curves 1, 2, and 3 represent oxygen free, 18% oxygen, and fully saturated with oxygen conditions in a batch reactor, respectively, while curves 1′, 2′, and 3′ indicate the same oxygen levels in a semi-batch reactor. The solid curves correspond to batch reactor, and the dashed curves correspond to the semi-batch reactor. Reprinted/adapted with permission from Ref. [63]. Copyright 2022, American Chemical Society.

**Figure 20:**
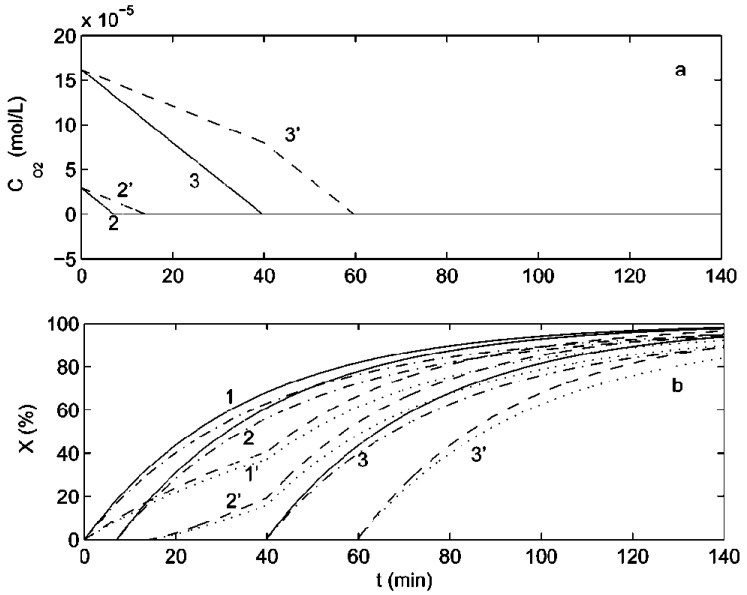
Comparison of (**a**) oxygen and (**b**) monomer conversion profiles in a batch and a semi-batch reactor at three oxygen levels and two MEHQ levels (0 and 200 ppm). Curves 1, 2, and 3 represent oxygen free, 18% oxygen, and fully saturated with oxygen conditions in a batch reactor, respectively, while curves 1′, 2′, and 3′ indicate the same oxygen levels in a semi-batch reactor. The solid curves correspond to the batch reactor, and the dashed curves correspond to the semi-batch reactor. The solid, dashed, dashed-dotted, and dotted curves correspond to batch without MEHQ, semi-batch without MEHQ, batch with MEHQ, and semi-batch with MEHQ processes, respectively. Reprinted/adapted with permission from Ref. [63]. Copyright 2022, American Chemical Society.

To compare the inhibition performance of oxygen in AA polymerization, Li et al. performed simulations in batch and semi-batch processes for three cases: oxygen free, 18% oxygen, and fully saturated oxygen [63]. It is noteworthy that in a semi-batch reactor, oxygen and other species are ingested slower than in a batch reactor, notwithstanding the fact that in a semi-batch reactor, oxygen is injected into the reaction environment continuously; even if the oxygen level is 18% in the feed, its concentration vanishes to zero before the completion of the feed interval (see Figure 19a). A semi-batch reactor has a longer induction time at the same oxygen level because of its slow dynamics. A slow flow rate induces a long induction time. It is evident from Figure 19b that the discontinuation of feeds causes the conversion to increase faster for the case of 18% oxygen level. It can be inferred from the simulations of Li et al. for a batch process which employs the same parameters as derived from the experimental data of Cutie et al. [68] that no synergism is shown in the inhibition performance of MEHQ and oxygen; however, the polymerization becomes retarded. Similar conclusions can be drawn for a semi-batch reactor if the same kinetic parameters are employed. Figure 20 depicts the influence of MEHQ on the oxygen concentration and monomer conversion at a typically employed commercial MEHQ concentration of 200 ppm with other parameters being the same as those in Figure 19. The oxygen concentration is not influenced by MEHQ; however, the monomer conversion becomes retarded by it. Additionally, they found that the strength of retardation does not significantly depend on the type of reactor employed.

**Figure 21:**
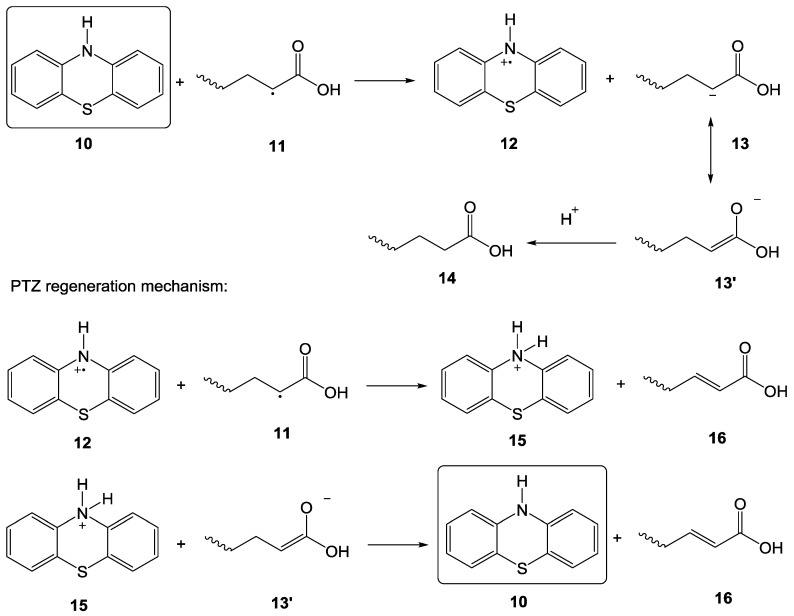
PTZ inhibition mechanism of acrylic acid polymerization [54].

Another important inhibitor is PTZ which is a highly efficient, commercially employed liquid-phase inhibitor that can work in both oxygenated and oxygen-less environments [69]. PTZ works as an amine-radical trap in an aprotic nonpolar solution and possesses an efficacy of two radical neutralization per PTZ molecule [70]. However, in an acrylic acid medium, PTZ acts via a catalytic mechanism. Levy et al. studied the inhibition mechanism of PTZ [67], as shown in Figure 21 [54]. The efficiency of PTZ (10) is independent of oxygen level, since it rapidly reacts with propagating chains as compared to oxygen [66]. The inhibition mechanism of AA polymerization by PTZ is as follows: (a) PTZ becomes oxidized to a radical cation (12); (b) the carbon-centered radical (11) transforms to carbanion (13); (c) (13) is further neutralized to 14; (d) radical cation (12) scavenges a hydrogen atom from (11) resulting in the formation of acrylic acid derivative (16) and protonated PTZ (15); and (e) the anionic intermediate (13′) combines with (15) to make it lose proton with the regeneration of PTZ. It is noteworthy that due to the regeneration of PTZ during the inhibition of the polymerization reaction, the inhibition capacity of PTZ lasts for a long time.

**Figure 22:**
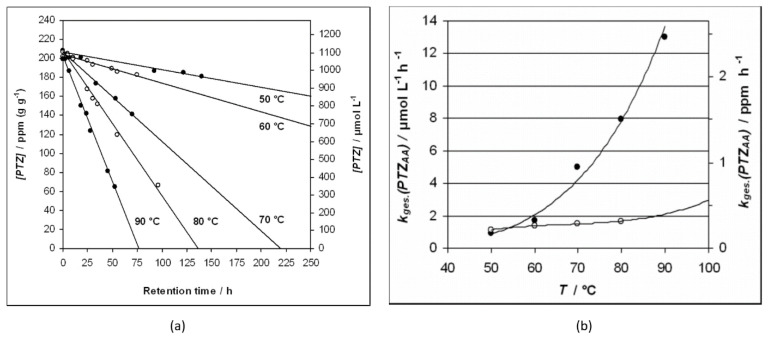
(**a**) Variation of PTZ concentration with the retention time at various temperatures in acrylic acid medium, and (**b**) rates of PTZ consumption in acrylic acid as a function of reaction temperature in air (solid circles) and N_2_ environment (open circles). Reprinted/adapted with permission from Ref. [71]. Copyright 2022, John Wiley and Sons.

Becker et al. examined the PTZ consumption in an acrylic acid medium via the die measurement technique [71]. They investigated the consumption kinetics under an air (21 vol.% O_2_) and under a nitrogen (0 vol.% O_2_) environment. Their results exhibited a linear decrease in PTZ concentration with increasing retention time, as displayed in Figure 22a. The PTZ consumption rates in acrylic acid under the air atmosphere were found to be higher than that under the nitrogen atmosphere. The consumption rates of the linear consumption curves in the temperature range from 40 to 100 °C is shown graphically in Figure 22b. 

## 5. Conclusions

Notwithstanding the existing knowledge in the process, plant design, and the availability of commercial equipment to control spontaneous and self-sustaining polymerization reactions, accidents continue to happen. This ignorance calls for urgent attention from the scientific community to focus their research on developing polymerization inhibition techniques since they offer a feasible and promising alternative method that can be incorporated, either alone and/or with emergency relief venting and secondary cooling. Additionally, due to the increasing pressure on industries to reduce environmental emissions, reaction inhibition which is a total containment technique is highly commended. In this review, we presented a thorough overview of the recent trends and advancements in the development of polymerization inhibition techniques. Polymerization inhibition techniques turn out to be a good method that can successfully terminate the runaway mechanism and precisely control the process, if used in properly tuned conditions. The primary goal of the researchers should be to determine the required inhibitor concentration that can allow an adequate induction period for the plant operator to implement safety measures before the occurrence of a secondary runaway. In addition, a proper injection and mixing system based on viscosity measures should be designed to provide a homogeneous inhibition in the system. Moreover, the inhibition system should be robust so that it can be employed in any type of industrial setting where the reactive monomers are stored or handled in bulk, suspension, or emulsion form. 

The various common types of radical inhibitors utilized for the inhibition of monomer polymerization in industries along with their advantages and disadvantages are tabulated in Table 7. Amongst the various available inhibitors, TEMPO and its analogues are of prime importance due to their high efficacy in inhibiting styrene polymerization. The DFT calculations have demonstrated a strong direct proportionality relationship between the electrophilicity of the TEMPO analogues and their efficacy to inhibit polymerization reaction. It is surmised that the solubility of the TEMPO analogues in toluene and the keto–enol tautomerism of some molecules, such as oxo-TEMPO, play a crucial role in inhibiting styrene polymerization in toluene. Moreover, 4-hydroxy-TEMPO and 4-oxo-TEMPO are found to be best anti-polymers, while BHT and DTBMP are the best antioxidant inhibitors. DTBMP when used alone produced the lowest growth percentage of 16.4 after 4 h operation duration.

Moving towards the synergistic effect, the combination of DTMBP (75 wt.%) with 4-hydroxy-TEMPO (25 wt.%) exhibited the most significant inhibition effect yielding the lowest growth percentage of 8.60 after 4 h operation duration. DBHA is also found to be an excellent inhibitor for styrene polymerization but only in an oxygenated environment. The peroxyl radicals effectively abstract hydrogen from DBHA and terminate the propagation chains. Nevertheless, in the absence of oxygen, hydrogen abstraction becomes difficult due to the increase in the number of oligostyryl radicals and hence the inhibition mechanism becomes ineffective. MEHQ is a standard inhibitor that is used in industry to inhibit spontaneous polymerization of acrylic acid. The dissolved oxygen in the reaction system provides a synergistic effect to MEHQ and enhances its inhibition effect under the production conditions of acrylic acid. 

## Figures and Tables

**Table 1:** Accident data pertaining to styrene and other chemicals in USA, Europe, and Asia since 1998 [11].

Date	Location	Fatalities	Injuries	Hazard	Chemical(s)
06/27/1998	Channahol, IL, USA	0	1	1. leakage	1. ethylbenzene 2. styrene
06/23/1999	Pasadena, TX, USA	2	4	1. fire 2. explosion	1. styrene 2. butadiene
07/25/1999	Hong Kong, China	0	0	1. explosion	1. styrene 2. trichloroethylene
09/13/1999	Newton, MA, USA	0	22	1. leakage	1. styrene
02/05/2000	Hong Kong, China	0	0	1. leakage	1. styrene
03/14/2000	Fredericton, NB, Canada	0	0	1. leakage	1. alcohol 2. styrene
03/27/2000	Pasadena, TX, USA	1	71	1. leakage 2. fire 3. explosion	1. butadiene 2. Cyclohexane 3. styrene
10/31/2000	Channel Islands, France	0	0	1. leakage	1. isopropyl alcohol 2. methyl ethyl ketone 3. styrene
04/04/2001	Zhejiang, China	0	0	1. leakage	1. styrene
04/17/2001	Shanghai, China	0	0	1. leakage	1. styrene
10/29/2001	Marietta, OH, USA	0	0	1. leakage	1. styrene
02/13/2003	Hangzhou, China	0	0	1. leakage	1. styrene
03/12/2003	Yeochon, Republic of Korea	1	0	1. explosion	1. styrene
04/08/2004	Jiangsu, China	6	8	1. leakage	1. styrene
06/07/2004	Canada, USA	0	0	1. leakage	1. styrene

**Table 2:** Thermal runaway incidents associated with styrene processing in various parts of the world [11,12,13,14,15,16].

Date	Location	Consequences	
		Injury	Fatality
01/21/1998	Kaohsiung, Taiwan	4	0
12/24/1998	Kanagawa, Japan	0	0
06/27/1998	Channahon, IL, USA	1	0
06/23/1999	Pasadena, TX, USA	21	2
10-06-1999	Chiayi, Taiwan	1	0
03/27/2000	Pasadena, TX, USA	71	1
04-02-2003	Addyston, OH, USA	0	1
04-08-2004	Jiangsu, China	8	6
06/30/2005	Mesa, AZ, USA	0	1
07/11/2006	Mainland China	Only Equipment Damage	
03/05/2008	Mainland China	Only Equipment Damage	
21/09/2014	Fairfield, AL, USA	1	2
06/02/2017	Taiwan	4	0
29/01/2018	Taiwan	Only Equipment Damage	
07/05/2020	Visakhapatnam, India	585	13
14/04/2022	Andhra Pradesh, India	12	6

**Table 4:** Values of global chemical potential (μ^o^), chemical hardness (η^o^), and electrophilicity (ω^o^) obtained from DFT calculations [50].

Inhibitor	Global Chemical Potential (μ^o^)	Chemical Hardness (η^o^)	Electrophilicity (ω^o^)
BHT	−2.6654	2.9900	1.1880
TBC	−3.2072	2.7878	1.8448
TBHQ	−3.2376	2.6937	1.9457
DTBMP	−2.5161	2.8021	1.1297
MEHQ	−3.2282	2.6478	1.9680

**Table 5:** The weights of polymer, the growth percentage, and the conversion percentage of styrene in the presence of phenolic inhibitors after 4 and 8 h of operation [50].

After 4 h of Operation						
Inhibitor	Weight (g)	Growth Percentage	Outlet Mass Fraction (wt.%)			Conversion (%)
			Styrene	Dimer	Trimer	
BHT	0.285	42.50	99.839	0.022	0.010	0.111
TBC	0.305	52.65	99.811	0.028	0.012	0.139
TBHQ	0.363	81.25	99.749	0.034	0.015	0.201
DTBMP	0.233	16.40	99.902	0.012	0.005	0.048
MEHQ	0.387	93.35	99.730	0.053	0.023	0.251
**After 8 h of Operation**						
BHT	0.399	99.50	99.713	0.036	0.015	0.237
TBC	0.470	135.08	99.643	0.038	0.016	0.307
TBHQ	0.526	162.81	99.568	0.054	0.023	0.382
DTBMP	0.319	59.47	99.812	0.019	0.008	0.138
MEHQ	0.630	215.16	99.493	0.062	0.027	0.491

**Table 6:** Effect of concentration of quinones and amines on the rate of polymerization of MMA. (r) in dark mode. Other parameters are (AIBN) = 1.14 × 10^−2^ mol/L and temperature = 60 °C. Reprinted/adapted with permission from Ref. [59]. Copyright 2023, Pleiades Publishing, Ltd.

Inhibitor Mixture	(BQ)] × 10^3^, mol/L	(Amine), mol/L	r × 10^4^, mol/(l s)
BQ-1–DMA	4.25	0.08	2.8
	4.25	0.21	2.8
	4.25	0.425	2.8
	2.12	0.21	3.0
	3.19	0.21	3.0
	8.5	0.21	2.5
BQ-1–DMPA	4.25	0.08	2.1
	4.25	0.21	2.1
	4.25	0.425	2.1
	2.12	0.21	2.6
	3.19	0.21	2.6
	8.5	0.21	1.8
BQ-2–DMA	4.25	0.425	3.0
BQ-3–DMA	4.25	0.425	3.0
BQ-4–DMA	4.25	0.425	3.0
BQ-5–DMA	4.25	0.425	2.7

**Table 7:** Common types of free radical inhibitors used in industries to inhibit monomer polymerization.

S. No.	Free Radical Inhibitor	Monomer	Advantages	Disadvantages
1.	TEMPO and TEMPO-derivatives	Ethylene, Butadiene, Vinyl monomers, and MMA.	1. High efficacy. 2. Cheap cost. 3. High stability.	1.It shows inhibitory properties at high concentration. 2. Toxic.
2.	4-methoxyphenol (MEHQ)	Styre1ne, Acrylic Acid.	1. Stable at higher temperatures.	1. Not effective in the absence of oxygen.
3.	Phenothiazine (PTZ)	Acrylic Acid (AA).	1. Highly effective for AA. 2. Effective even in absence of oxygen. 3. Highly efficient.	1. Toxic.
4.	Hydroquinone (Hq)	Methyl methacrylate (MMA), Vinyl acetate, Acrylic Acid.	1. Oxygen-independent inhibitor.	1. Toxic.
5.	Ortho-benzoquinone	Methyl methacrylate (MMA).	1. Oxygen-independent inhibitor.	1. Toxic.
6.	N,N-dimethylaniline (DMA)	Methyl methacrylate (MMA).	1. Stable at high temperatures.	1. Quite expensive.
7.	N,N-dimethylisopropanolamine (DMPA)	Methyl methacrylate (MMA).	1. Less corrosive.	1. Toxic.
8.	4-tert-butylcatechol (TBC)	Styrene, Butadiene.	1. Easy to remove prior to polymerization by alkalinewashing, by distillation, or by passing through an activated alumina column.	1. Has low vacuum pressure and hence in gaseous processes.
9.	Tert-butyl hydroquinone (TBHQ)	Styrene, Butadiene.	1. Stable at high temperatures. 2. Non-toxic. 3. Does not cause discoloration.	1. Fire hazard.
10.	2,6-di-tert-butyl-4-methoxyphenol (DTBMP)	Styrene.	1. Easily handled liquid product. 2. Stable at higher temperatures.	2. Toxic.
11.	2,6-Di-*tert*-butyl-4-methylphenol (BHT)	Styrene, Butadiene. MMA, Acrylic Acid.	1. Non-toxic.	1. Fire hazards.
12.	N,N-Dibenzylhydroxylamine (DBHA)	Styrene.	1. Non-toxic.	1. Causes eye and skin irritation. 2. Not efficient in the absence of oxygen.
13.	2,5-Di-tert-butyl-hydroquinone (DTBHQ)	Styrene.	1. Highly effective and widely used in industrial processes.	1. Highly toxic.
14.	2,4-dinitrophenol (DNP)	Styrene.	1. Effective even in absence of oxygen.	1. Toxic. 2. Fire hazard.
15.	2,4-dinitro-6-sec-butyl phenol (DNBP)	Styrene.	1. Effective even in the absence of oxygen.	1. Toxic.
16.	2,6-Dinitro-p-cresol	Vinyl aromatic monomers, such as styrene monomer.	1. Stable polymerization inhibiting performance. 2. Low unit consumption. 3. Low toxicity.	It is solid and can become unstable if subjected to temperatures above its melting point and may explode.
17.	Phenylenediamines	Vinyl aromatic monomers, such as styrene monomer.	1. Non-toxic.	1. Ineffective in the absence of oxygen.
18.	Aryl amines	Styrene.	1. Efficient even at reduced concentration.	1. Fire hazard.
19.	p-Benzoquinone	Styrene, Acrylic Acid, Methyl methacrylate.	1. Oxygen-independent inhibitor.	1. It is very difficult to obtain color-free monomers when they have been inhibited with p-benzoquinone.
20.	Oxygen	Styrene,Methyl methacrylate.	1. Easily available. 2. Cheap.	1. Needs instrumentation to control the amount of flow.

## Data Availability

Not Applicable.
